# Developing an Open Innovation Attitude Assessment Framework for Organizations: Focusing on Open Innovation Role Perspective and Locus of Activity

**DOI:** 10.3390/bs12020046

**Published:** 2022-02-11

**Authors:** YoungPyo Jun, Kilsun Kim

**Affiliations:** 1 Sogang Business School, Sogang University, Seoul 04107, Korea; kilsunkim@sogang.ac.kr; 2 KPMG Consulting, Seoul 06236, Korea

**Keywords:** open innovation, organizational open innovation attitude, open innovation role perspective, open innovation brokerage, open innovation locus of activities

## Abstract

From an organizational perspective, open innovation (OI) capability assessments are becoming increasingly important. The authors propose that an organization’s attitude toward interactive OI activities among OI stakeholders can reveal its degree of capability. This paper aims to focus on an organization’s OI attitude measurement scales and develop a framework linked to the role perspectives and loci of OI activities occurring at the organizational level. This research will introduce a practical, theory-based indication of OI assessment by combining a deductive process that identifies organizational OI attitude constructs with an inductive framework development process. First, the authors conducted an extensive literature review of attitude measurement on the execution of OI. Then, they performed empirical data analysis using a large-scale structured attitude assessment survey from individuals in domestic and multi-national corporations (n = 134), which led to the development of questionnaire sets on attitude evaluation. This study contributes to developing an organizational OI attitude assessment scale. Furthermore, based on empirical data analysis, the research framework demonstrated the reliability and validity of the organizational OI attitude measurement scale. Specifically, the scale contains proven questionnaires assessing OI attitudes by interrogating individual actors’ impact, behavior, and cognition regarding their organization’s OI activities. The organization’s three role perspectives (transfer, absorption, and brokerage) and two loci of activities in the OI ecosystem provide six distinct dimensions, suggesting areas of focus for a firm’s strategic OI direction.

## 1. Introduction

Organizational capabilities for executing open innovation (OI) are growing quickly. Likewise, corporate executives are dealing with generating and managing innovative resources as they become increasingly sophisticated from an industry–ecosystem standpoint. As such, the nature of corporate innovation is undergoing a fundamental transformation, with far-reaching implications for both internal and external corporate areas. OI is a new method that profoundly challenges the traditional approach to innovation management and, lately, has emerged as one of the hottest topics in management science [1,2]. Chesbrough [3] introduced the open innovation concept, saying that “[…] open innovation is a paradigm that assumes that firms can and should use external ideas as well as internal ideas, and internal and external paths to market as the firms look to advance their technology” (p. xxiv).

Sivam et al. [4] examined the factors influencing a firm’s capacity to embrace and practice open innovation. Their study suggested that conditions such as culture, leadership, and strategy are the main drivers of an OI arena, highlighting culture as the most important. As Podmetina et al. [5] demonstrated in developing a competency model for OI, open, collaborative innovation is an essential area of process and expertise. In a similar vein, the ability to work in an interdisciplinary environment is key. Podmetina et al. [5] identified the individual-level capabilities needed to adopt OI, and accordingly, they developed an organizational capability model for open innovation from a human resource management perspective.

The assessment and management of an organization’s OI activities are highly important for managers and researchers to make sense of the ongoing interactive activities in the OI ecosystem. Hence, there is a pressing need for well-grounded measures of corporate-level OI capabilities in general. However, there has been little scholarly research on corporate-level assessments that evaluate the readiness of corporations for participation in such an environment [6], therefore necessitating a more systematic investigation of OI activity modes and measurement at the organizational level. 

In this research, these previously defined organizational OI capabilities are used as a basis for organizational OI attitude measurement criteria. This paper focuses on psychometric reflections on current OI activities performed by innovation actors, which can reveal the status of the organization’s OI capabilities. 

This research framework is especially valuable because the increasing demand for firms pursuing innovative outcomes through efficient capability management is germane not only to identifying organizational attitudes but also to measuring them at the organizational level. Ultimately, an organization-level OI attitude measurement and evaluation tool applicable to business practices will be highly useful in encouraging the general acceptance of open innovation theory as a practical innovation methodology. 

**Figure 1:**
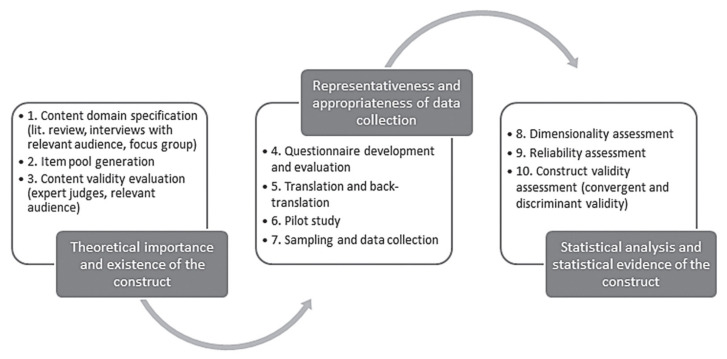
Ten steps and three phases in scale development (Slavec and Drnovesek, 2012 [7]).

The researchers of this study streamlined the methodology described by Slavec and Drnovesek [7] to assess the procedures used to develop a measuring instrument. As illustrated in Figure 1, they introduced ten steps grouped into three phases: “(1) the theoretical importance and existence of the construct, (2) the representativeness and appropriateness of data collection, and (3) statistical analysis and statistical evidence of the construct” [7]. 

Based on a thorough theoretical and empirical framework design, the contribution of this study is twofold. First, this research proposes a genuine, empirical evidence-based firm-level OI attitude assessment framework. Second, a new tripartite classification of the roles in the OI ecosystem is introduced. Measuring OI attitude by segmenting it into the roles of transfer, absorption, and brokerage will provide an assessment tool that can be applied more practically. Indeed, the results of the empirical data analysis were highly encouraging. A structured questionnaire was applied based on the organization-level OI attitude assessment framework. The statistical analysis of the collected data verified the usefulness of the method as well as the scale’s reliability and validity.

This paper first introduces an overview of the literature on measurement scale development and organizational OI activities. It then discusses the framework of open innovation attitude assessment at the organizational level. The following sections deal with data collection and statistical analyses to verify the reliability and validity of the framework. Finally, the study concludes with the attitude assessment model’s potential applications and avenues for future research.

## 2. Materials and Methods

### 2.1. Literature Review

Literature review for various subject areas required for the research procedure is explained as follows. 

*Attitude as a Measurement Component for a Behavioral Predictor*. “An attitude is a learned predisposition to behave in a consistently favorable or unfavorable way with respect to a given object” [8]. It is also defined as “a mental and neural state of readiness organized through experience, exerting a directive or dynamic influence upon the individual’s response to all objectives and situations with which it is related” [9]. In practice, terms such as values, judgments, beliefs, emotions, opinions, or intentions are used interchangeably with the term “attitude” [10,11].

Specifically, Davis et al. [12] compared two theoretical models regarding user acceptance of a certain object: computer technology. According to the theory of reasoned action (TRA), a widely studied model from social psychology, a person’s performance of a specific behavior is determined by their behavioral intention (BI) to perform the behavior, which, in this research, refers to open innovation activities. As shown in equation (1), a particular BI is jointly determined by the person’s attitude (A) and subjective norm (SN) concerning the behavior in question [13,14].

BI = A + SN(1)

Based on the definition of attitude as “an individual’s positive or negative feelings about performing the target behavior” [14], TRA suggests that a person’s attitude toward a behavior is determined by one’s salient beliefs ($b_{i}$) about the consequences of performing the behavior multiplied by the evaluation ($e_{i}$) of those consequences:

(2) $$A=\sum b_{i}e_{i}$$

Similar to TRA, the technology acceptance model (TAM) hypothesizes that computer usage is determined by BI, but TAM differs from TRA in that BI is viewed as being jointly determined by the person’s attitude toward using the system (A) and the system’s perceived usefulness (U), with relative weights estimated by regression:

BI = A + U(3)

**Figure 2:**
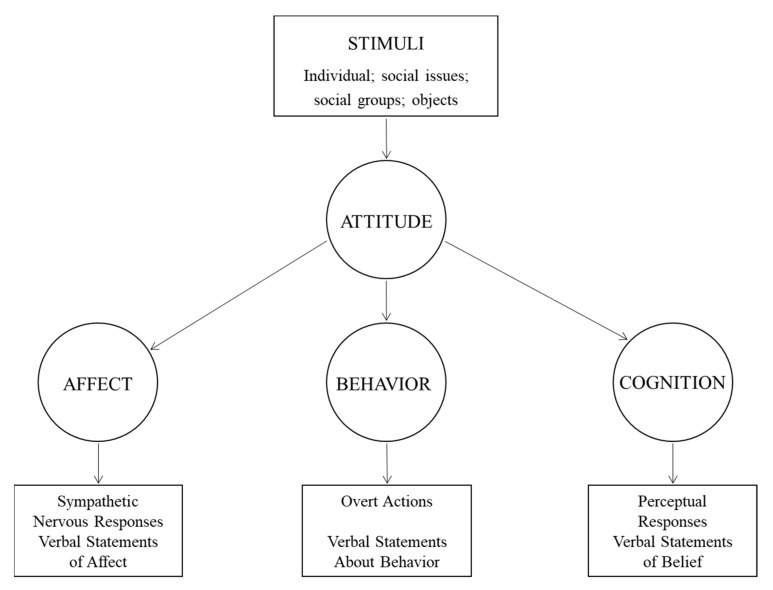
ABC model of attitude (Breckler, 1984 [16]).

When examining attitudes toward a behavior, each belief links the behavior to a certain outcome or to some other attribute such as the time invested in performing the behavior [15]. Thus, people automatically and simultaneously acquire an attitude toward a behavior because the attributes linked to the behavior are already valued positively or negatively. In this manner, people learn to favor behaviors believed to have primarily desirable consequences and form unfavorable attitudes toward behaviors associated with predominantly undesirable consequences. Specifically, the outcome’s subjective value contributes to an individual’s attitude in direct proportion to the strength of the belief, that is, the subjective probability that the behavior will produce the outcome in question. According to the tripartite (ABC) model of attitude [16], an attitude is composed of three components: affective, behavioral, and cognitive (see Figure 2). The affective component deals with feelings or emotions that are elicited by the stimuli. 

Results of previous studies have generally supported the hypothesized relationship between attitudes and behavioral intentions leading to actual behavior [17,18,19,20,21]. Regarding the scholarly works previously reviewed, this study’s attitude assessment survey questionnaire focuses on measuring the level of perceived belief in certain OI behavioral activities by individuals within an organization. As such, a measured degree of attitude can be interpreted as the level of intention to perform OI activities in the present.

Table 1 lists references for organizational OI capabilities divided into categories, demonstrating how organizational OI capabilities are defined and linked to the organizational OI attitude. The organizational OI capabilities are categorized as market sensing, building or possessing networks and processes for collaboration, and maintaining a corporate culture that encourages and rewards the OI activities leveraged by motivated leadership. These activities are used in the following section to construct OI attitude assessment questionnaires. 

*Classification of Role Perspective and Locus of Activity in the OI Ecosystem*. Research regarding open innovation embraces a broad range of themes such as roles, types, dimensions, competencies, and capabilities, studied using various perspectives including the organizational level, functions, and partnerships. As such, OI has developed as a model that connects the macro and micro levels in innovation studies [2,34], in which companies strive for innovation partly by tapping into knowledge that exists outside their organizational or market boundaries and partly by allowing their own internally developed knowledge to flow outward for external use [35].

*The Classification of Role Perspectives.* Several researchers have applied three core OI processes in their research [24,36,37]. Subsequently, they commonly categorized OI activities into three modes based on whether the direction of the OI activities was inside-out (outbound), outside-in (inbound), or coupled. From a functional perspective, it is also possible to distinguish the roles of exploration, exploitation, and ambidexterity in an open innovation ecosystem [38,39,40,41].

While the authors mentioned above discussed the processes or functions of OI activities, this paper focuses on assessing the OI attitudes of an organization in an ecosystem to evaluate its current status pertaining to OI performance. Thus, this paper investigates the role of firms in the market, whereas previous literature focused on the flow of knowledge. Therefore, the perspective-oriented approach is more appropriate for measuring attitudes at the firm level. During the research, the attitude measurement questionnaire was composed according to an organization’s position from the role perspectives: transfer, absorption, and brokerage. 

One of the peculiarities of this paper is the recognition that the role of the intermediary in the OI ecosystem shares the same axis with two other roles. Consequently, in this paper, the roles of intermediary and broker are synonymous, and the authors intend to unify them by using the term “brokerage.”

Previous studies have highlighted the role of brokerage in innovative environments. For example, Howells [42] argued that intermediaries might be key players in the transformation from closed to open modes of innovation. Similarly, Stewart and Sampsa [43] explained that innovation intermediaries are persons or organizations that facilitate innovation by linking multiple independent players to encourage collaboration and open innovation, thus strengthening the innovation capacity of firms, markets, industries, or countries. Additionally, many authors have described brokers [44,45,46]. In particular, Gould and Fernandez [45] generated detailed insight into brokerage behavior by describing it as the facilitation of information flows with or without a direct reward. They argued that the various interests of actors would affect the way they seize brokerage opportunities. From an organizational behavior perspective, it is critical to confirm that the broker plays an important role on a team, particularly in the OI ecosystem. Regarding cultural brokerage, Jang [47] illustrated that when people from diverse cultures are called to work together, they do not navigate their differences in isolation. Rather, individuals with multicultural backgrounds often emerge as cultural brokers and help their monocultural counterparts, positively influencing team performance. 

In the context of OI, brokers facilitate interactions among firms by providing an appropriate architecture to create and capture external networking opportunities [24,37,48,49,50]. In general, OI brokers mediate both inbound and outbound OI. Thus, OI brokers can reduce internal fear of experimentation, guiding processes of business model selection, R&D, finding suitable partners, and the deployment of internal sources to external markets by providing access to a broader repository of ideas and technologies [28,51,52,53]. 

Table 2 below distinctively presents organizational perspectives on roles in the OI ecosystem.

*Dual Loci of Organizational Activity Scope.* In addition to three role perspectives, a great number of studies distinctively categorize OI roles at the intra- and inter-organizational levels [24,36,86,87]. On the intra-organizational level, OI activities within a firm are associated with inner-company tasks and cooperation among stakeholders. Meanwhile, inter-organizational-level activities are primarily associated with external OI networks for collaboration. 

Researchers such as West and Bogers [37] have discussed leveraging external sources for internal use in innovation. Lassen and Laugen [88] also explored the influence of in/external collaboration on the degree of newness in OI. Therefore, it is reasonable to interpret internal and external measurements separately. Comparing the OI capabilities of various organizations within an ecosystem necessitates using a different scope of evaluations beyond organizational, which the division of loci enables. On the other hand, the national and international scopes of OI activities are compared by Clauss and Spieth [89]. Based on previous literature, the loci of OI activities take place according to organizational activity scopes, as shown in Table 3 below.

### 2.2. Research Framework and Scale Development

The conceptual research model for assessment of the organizational OI attitude and the explanation of the validation process for measurement item are as follows. 

#### 2.2.1. Conceptual Research Model for Organizational OI Attitude Assessment Framework

In this research framework, organizational OI attitudes constitute members’ collective state of mind within a specific organization. Individual innovation actors play a critical role in OI activities in intra- and inter-organizational settings and the OI ecosystem as transferers, absorbers, and brokers. Therefore, organizational OI attitudes can be measured through the set of individual OI attitudes.

Bock et al. [104] examined the theoretical framework for TRA and augmented it with extrinsic motivators, social-psychological forces, and organizational climate factors believed to influence individuals’ open innovation attitudes, such as knowledge sharing. They confirmed the hypothesis that attitudes toward and subjective norms regarding knowledge sharing as well as organizational climate affect individuals’ intentions to share knowledge.

An organization’s attitude toward open innovation is a pre-stage or part of its capabilities. The sections of the questionnaire are categorized based on the open innovation activity criteria at the organizational level to measure the organization’s attitude toward open innovation.

**Figure 3:**
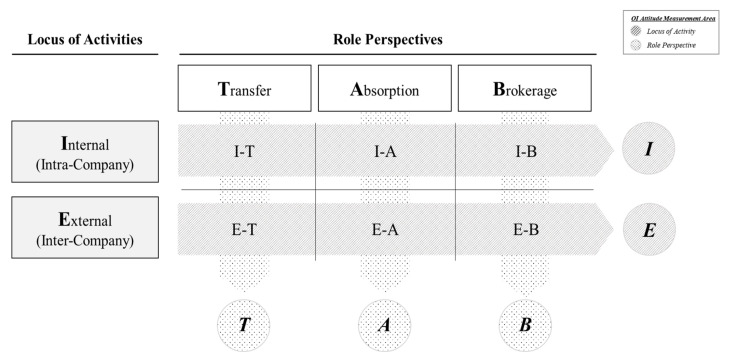
Conceptual research model for organizational OI attitude assessment framework (authors’ own work).

This research originated from the confidence that an assessment tool for measuring organizational OI attitudes will be useful for those who pursue OI. By examining OI from the organization’s role perspectives and loci of activities, a firm would be able to recognize specific areas that need stronger support for OI activities. In Figure 3 below, a 2×3 matrix of the loci of activities and role perspectives in OI ecosystems is proposed to evaluate attitudes identifying specific areas of organizational OI needs. 

#### 2.2.2. OI Attitude Assessment Questionnaire Development and Validation

Psychometricians assert that the validity of a measurement scale is built-in from the outset. Following Nunnally’s [105] suggestions, the authors focused on ensuring the validity of the plan and procedures for construction rather than testing the validity of measures after they were constructed. Content validity, defined as “the degree to which the score or scales being used represent the concept about which generalizations are to be made” [106], was established by carefully selecting the initial scale items. In discussing content validity, psychometricians often appeal to the domain sampling model [105,106], which assumes that there is a domain of content corresponding to each variable one is interested in measuring; candidate items representative of the domain of content are selected and measured to obtain generalizable results. In this method, researchers should begin by articulating conceptual definitions of what is to be measured and preparing items to fit the construct definitions [107].

In this research, the OI attitude construct refers to an organization’s attitude toward executing OI activities to accomplish goals within a firm and an industry ecosystem. The construct will be interpreted as the perceived state of mind resulting from an organization’s real open innovativeness status that collectively measures a company’s readiness to execute an innovation outcome. As discussed above, several researchers have identified specific beliefs and motivations that may enhance the adoption of certain objectives, such as new technologies [12,108].

#### 2.2.3. Exploration of the Concepts of Content Validity and Item Generation

The development of the corporate open innovation attitude assessment (COIAA) scales consisted of an explorative and a confirmative analysis. The objective of this phase in developing the COIAA scale was to define the concept of the organizational open innovation attitude and to generate items. This phase consisted of Hollis reliability tests, item-total analysis, explorative analysis, and confirmatory analysis.

This study’s statistical analysis went through the following four steps. First, it followed a procedure for deriving COIAA construct assumptions by performing Hollis reliability analysis on expert surveys. Next, a pilot survey was conducted to extract factors for all 60 items of the expert survey through item analysis. Following the previous two steps, the final results of this study were presented through exploratory and confirmatory factor analysis.

#### 2.2.4. Hollis Reliability Test for Delphi Survey (1)

In this qualitative study, the first Delphi survey was conducted by designing an open-ended questionnaire targeting experts in business, the public sector, and academia related to open innovation. The experts were carefully selected from the recommended candidates in business and academic fields through the request to the Korean Management Association. The Hollis reliability test was performed to ensure the validity of the first Delphi study to measure the reliability of the structure and factors of the survey. A total of six experts were selected, with two people each from business, the public sector, and academia with a high level of experience and understanding of open innovation practices in organizational contexts. 

The Hollis reliability coefficient for Delphi Survey (1) estimated the expert’s consistency and consensus on the structure and factors of the research model. The higher the degree of expert consensus, the more objectivity-supported are the reliability and validity of factors related to the required stage (input stage, process stage, output stage, and effect stage) of the specialized project diagnosis model to which the integrated evaluation model is applied.

The Hollis reliability coefficient for Delphi Survey (1) is measured as follows:

Hollis Reliability Coefficient = 

n1: assessor 1,

n2: assessor 2,

M = number of matches between Respondents 1 and 2

The results of Delphi Survey (1) are as follows: regarding the OI locus of activities, the internal (intra) was ranked first and the external (inter) as second, respectively, followed by Joined and International [109,110]. As a result of examining OI role perspectives, Transfer: inside-out (outbound) was marked first, Absorption: outside-in (inbound) was ranked second, and Brokerage: coupled (by third party) was ranked third. Then, exploitation, exploitation, and ambidexterity followed, in that order [111]. 

In this research, two factors of OI locus of activities, Internal (Intra) and External (Inter), were selected based on the closed-ended Delphi survey questionnaire. OI role perspectives confirmed three factors: transfer, absorption, and brokerage. The results of the reliability and validity of Delphi Survey (1) are shown below. Overall, the reliability of Expert A and Expert B was 0.4000, that of Expert A and C was 0.4000, and that of Expert B and Expert C was 0.6000, which was estimated to be high (see Table 4).

The reliability analysis results of the evaluation indicators included in the evaluation areas of the request, input, process, output, and effect stages for the first Delphi survey are as follows.

(1) OI Locus of Activities.
  Expert A: B = 0.5000; Expert A: C = 0.5000; Expert B: C = 0.5000.
(2) OI Role Perspectives.
  Expert A: B = 0.3333; Expert A: C = 0.3333; Expert B: C = 0.6667.
(3) COIAA.
  Expert A: B = 0.4000; Expert A: C = 0.4000; Expert B: C = 0.6000.

#### 2.2.5. Hollis Reliability Test for Delphi Survey (2)

Unlike the first Delphi survey, the second Delphi survey was conducted by designing a closed-ended format targeting experts in business, the public sector, and academia related to open innovation. The Hollis reliability test was performed to measure the reliability of the structure and factors of the survey, ensuring the validity of the second Delphi study. A total of six experts were selected with two people each from business, the public sector, and academia with a high level of experience and understanding of open innovation practice in organizational aspect. 

The Hollis reliability coefficient for Delphi Survey (2) estimated the experts’ consistency and consensus on the structure and factors of the research model. The higher the degree of expert consensus, the more objectivity-supported are the reliability and validity of factors related to the required stage (input stage, process stage, output stage, and effect stage) of the specialized project diagnosis model to which the integrated evaluation model is applied.

The Hollis reliability coefficient for Delphi Survey (2) is measured as follows.

Hollis Reliability Coefficient = 2M/(n1 + n2)

n1: assessor 1,

n2: assessor 2,

M = number of matches between respondent 1 and 2

The results of Delphi Survey (2) are as follows: In terms of OI locus of activities, the internal (intra) and the external (inter) factors were taken for the second Delphi study. Based on the first Delphi survey results, Transfer: inside-out (outbound), Absorption: outside-in (inbound), and Brokerage: coupled (by third party) were selected for second Delphi study. 

In the second study, two factors of OI locus of activities—internal (intra) and external (inter)—and three factors of OI role perspectives—transfer, absorption, and brokerage—verified the reliability and validity of the first Delphi survey. The results of the reliability and validity of Delphi Survey(2) are shown below in the Table 5. 

Overall, the reliability of Expert A and Expert B was 0.4000, that of Expert A and C was 0.4000, and that of Expert B and Expert C was 0.6000, which was estimated to be high. 

The reliability analysis results of the evaluation indicators included in the evaluation areas of the request, input, process, output, and effect stages for the second Delphi survey are as follows:

(1) OI Locus of Activities.
  Expert A: B = 0.5000; Expert A: C = 0.5000; Expert B: C = 0.5000.
(2) OI Role Perspectives.
  Expert A: B = 0.3333; Expert A: C = 0.3333; Expert B: C = 0.6667.
(3) COIAA.
  Expert A: B = 0.4000; Expert A: C = 0.4000; Expert B: C = 0.6000.

#### 2.2.6. Results of the Pilot Study

Item analysis was performed on a total of 60 items to evaluate the items of the research tool for COIAA. For the pilot survey, the survey questionnaires were distributed online for those who were recommended by Korean Management Association. Fifteen respondents from academic, corporation, and entrepreneurship backgrounds were selected. As a result of analyzing 60 questions, the average score of the questions was 3.55 to 5.58, the standard deviation was 1.06 to 1.76, and the absolute value of the standardized values (Z-score) of skewness and kurtosis was 1.96 (*p* < 0.05). None of the above items appeared in Table 6. If the corrected item-total correlation between each item and all items is less than 0.30, the item is evaluated as having a low contribution within each scale area. It was verified as appropriate in the validity test for a total of 60 items used for item analysis in this study. The correlation coefficient between each item and all items was from 0.40 at the lowest to 0.76 at the highest, as shown in Table 7.

When an item is removed, the reliability coefficient (Cronbach’s alpha if the item is deleted) of 0.50 or more is considered as the minimum value, and in the case of studies that verified the construct validity, even 0.50 or more is considered acceptable. In this study, no items with a reliability coefficient of less than 0.50 were detected, and the reliability coefficient (Cronbach’s alpha if item is deleted) when an item was deleted was 0.97, indicating a high reliability coefficient. As a result of performing an exploratory factor analysis on a total of 60 items used in the pilot analysis, among which four items from IT07 to IT10, four items from IA07 to IA10, and four items from IB07 to IB10. Also, four items from ET07 to ET10, four items from EA07 to EA10, and four items from EB07 to EB10 were removed. Finally, a total of 24 items were removed.

#### 2.2.7. Item Analysis for COIAA Main Survey

As a result of finally analyzing 36 items to evaluate the items of the research tool for COIAA, the average score of the items ranged from 3.57 to 5.79, the standard deviation was 1.06 to 1.76, and the standardized values of skewness and kurtosis. There were no items with an absolute value of (Z-score) of 1.96 (*p* < 0.05) or higher (Table 8). If the corrected item-total correlation between each item and all items is less than 0.30, the item is evaluated as having a low contribution within each scale area. It was verified as appropriate in the construct validity test for the final 36 items used in the item analysis of this study. The correlation coefficient between each item and all items was 0.50 at the lowest and 0.79 at the highest. When an item is removed, the reliability coefficient (Cronbach’s alpha if the item is deleted) of 0.50 or more is considered the minimum value, and in the case of studies that verified the construct validity, even 0.50 or more is considered acceptable. No items with a reliability coefficient of less than 0.50 were detected in this study. Furthermore, the reliability coefficient (Cronbach’s alpha if the item is deleted) was found to be a minimum of 0.74 and a maximum of 0.95.

#### 2.2.8. Qualitative Research and the Generation of Initial Questionnaire Items 

For this research, the authors formulated a questionnaire measuring individuals’ perceived attitudes toward OI activities from three organizational role perspectives—transfer, absorption, and brokerage—and two loci of OI—internal and external. Previous studies have mentioned numerous aspects pertaining to these themes that formed the basis for generating scale items to measure an organization’s OI attitude. This basis was augmented with insights in the literature about attributes for assessing organizational attitudes in general. An initial pool of 36 OI attitude items was generated from this phase. Because an organization is a composition of individual members, the questionnaire was disseminated at the individual level. 

A procedural method was used to develop new multi-item scales possessing high reliability and validity. As stated above, the conceptual definitions of perceived open innovation capabilities consisted of transfer, absorption, and brokerage. The constructs of the OI questionnaire were derived through a literature review on components required at the organization level and consisted of areas that are generally perceived by members of the organization. In preparing candidate items, published research papers discussing an organization’s open innovation role perspectives and capabilities were reviewed to identify various facets of the constructs that should be measured [112]. 

When phrasing questionnaires, sentences should be written so that the sharpened syntax draws out the perceived reality of the respondent, who can easily understand the sentence using common sense. As defined earlier in this paper, the object to be assessed in this study is an organization’s attitude toward OI. An attitude is explained as “a relatively enduring set of beliefs, feelings, and behavioral tendencies toward socially significant objects, groups, events, or symbols” [113]. 

From a psychological perspective, assessed attitudes can be used to explain or articulate the underlying cause of observable behaviors or exhibited attitudes. As such, questions should be able to evaluate a particular entity with some degree of favor or disfavor [114]. As discussed previously, the tripartite model of attitudes describes three components consisting of affective, behavioral, and cognitive dimensions. While writing questions regarding OI attitude, it is necessary to ponder which intrinsic elements are functioning in each component. The affective component involves a person’s feelings or emotions toward an attitude object (e.g., I feel, I think). The behavioral component indicates how the attitude influences how a person acts or behaves (e.g., I would, I will, I intend to), and the cognitive component involves a person’s belief or knowledge about an attitude object (e.g., I believe). By applying these phrases to the organizational OI environment, the authors expect the OI behavioral intention of an organization to be consistent with the attitudes that people possess. 

The survey is composed of a questionnaire to identify commonsense attitudes toward OI. Respondents’ perceptions should reflect a certain degree of truth. In social science, the measurement of subjects by asking structured questions is a commonly used and accepted method of data collection. In one of the most notable cases, Parasuraman [115,116] adopted a multiple-item scale to measure subjects’ readiness to embrace new technologies. He constructed an item pool to assess customer perceptions about dealing with new technology to provide useful insights that can enhance the user experience in various ways.

The initial survey questionnaire was drafted based on a literature review on organizational OI activities and a study on the tripartite (ABC) model of attitudes. The ABC model breaks attitude down into three components and is used to reflect on an individual’s behaviors. The questionnaires are composed of verbs to measure each component of attitude. In order to measure the OI attitude of the organization to which the respondent belongs, the questionnaire focuses on feeling (affect), doing (behavior), and thinking (cognition) status [16].

The initial questionnaire was reduced, and phrases were modified based on feedback from a panel discussion. The panel was composed of a dozen academic and business associates in the OI field who evaluated the delivery of each question’s intended meaning. Next, the questionnaire was piloted among 15 individuals associated with the field of OI research and practice. Based on their feedback, those items that best fit the definitions of the constructs were retained, ultimately yielding six items for each construct. A structured pool of 36 corporate open innovation attitude assessment (COIAA) items was generated from this phase, after which the online survey was launched. Respondents were asked to answer each OI attitude item on a 7-point Likert scale, from *(1) strongly disagree* to *(7) strongly agree.* The final list of questionnaire items is presented in the Table A1.

## 3. Data Analysis Results and Discussion

In this section, the authors explain the process of the validation of constructs through data collection and analysis. 

### 3.1. Data Collection and Analysis 

The collection of data used in the study, the sample characteristics, and the analysis method are explained as follows.

#### Data Collection Process and Sample Description 

As mentioned previously, the structured questionnaire was prepared for personnel in all positions related to OI mechanisms in corporations, including SMEs and large firms, domestic and foreign. Respondent contact information is based on the database of the Korean Management Association. The survey questionnaires were sent out in form of via electronic mail. The cover letter described the survey’s objective, supplying Chesbrough’s [35] definition of OI to avoid possible bias due to different understandings of the concept. The online survey was launched in February 2020 and conducted until March 2020. Among the 700 surveys sent out, 151 people responded. The response rate was 22%. Finally, 134 samples were accepted for analysis, excluding eight insincere answers and nine with low understanding for the research subject. The descriptive sample statistics are shown in Table 9.

In general, scholars agree that larger sample sizes are likely to produce more stable correlations among variables and will result in greater replicability of exploratory factor analysis (EFA) outcomes. Velicer and Fava [117] found evidence that any ratio less than a minimum of three participants per item is inadequate. Moreover, there is additional evidence that factor saturation (the number of items per factor) and item commonalities are the most important determinants of adequate sample size [118,119]. 

When using EFA, Worthington and Whittaker [120] offered the following guidelines for scale development research sample sizes: “(a) sample sizes of at least 300 are generally sufficient in most cases, (b) sample sizes of 150 to 200 are likely to be adequate with data sets containing communalities higher than 0.50 or with 10:1 items per factor with factor loadings at approximately |.4|, (c) smaller samples sizes may be adequate if all communalities are 0.60 or greater or with at least 4:1 items per factor and factor loadings greater than |.6|, and (d) samples sizes less than 100 or with fewer than 3:1 participant-to-item ratios are generally inadequate” [120].

In this research, the subject of the survey regarding organizational OI activities and interactions is not intended for respondents from the general public. Rather, it is limited to those who have experience with and understand an organization’s role perspectives and the interactions between the internal and external scopes of OI activities. Though the sample size of 134 is less than the sample-size criteria of 150 addressed above, all the commonalities exceedingly satisfy the baseline of 0.6 or higher. In addition, the sampling adequacy was high, at 0.916, as shown in Table 10. 

**Figure 4:**
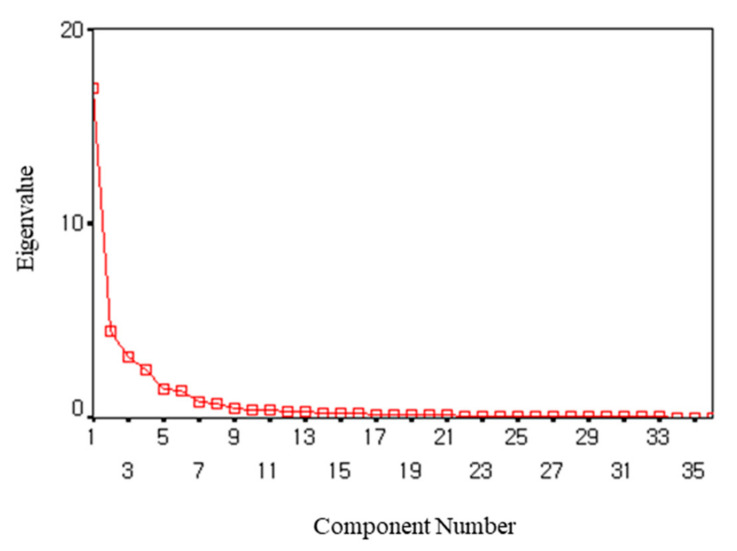
Scree plot for COIAA main survey.

**Figure 5:**
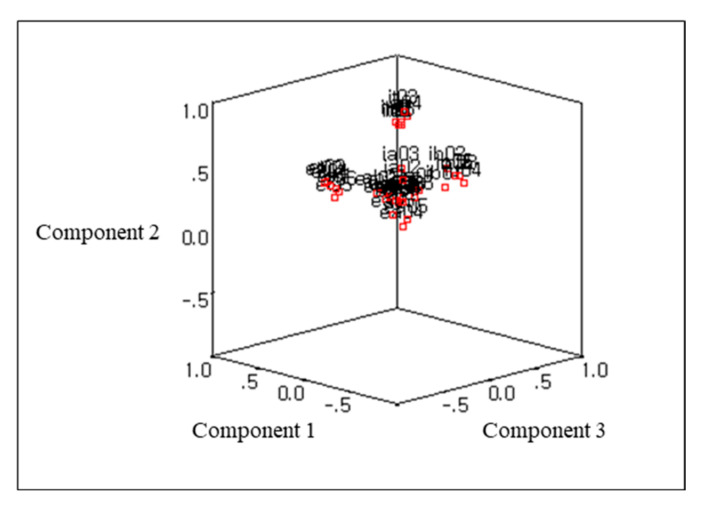
Component plot in rotated space.

The result of the KMO test to determine whether the variable coefficients and case coefficients of this study sample are suitable for factor analysis was 0.916 (KMO > 0.05). In order to check whether the correlation coefficient matrix is suitable for factor analysis, Bartlett, as a result of the sphericity test, χ^2^ = 6370.374 (*p* < 0.001), confirmed that the data were suitable for factor analysis. As for the factor extraction method, principal component analysis (PCA) was used to extract factors that explain as many parts as possible, and EFA was conducted by performing orthogonal rotation varimax. As a result of extracting factors using a total of 36 items, eight factors were extracted when an eigenvalue of 1.0 or higher was applied for each factor. Different methods for determining were applied in combination. First, in the case of the circle tool, it is composed of six factors, and in the Scree test, which is a method of showing the eigenvalues of each factor in Figure 4, it is confirmed that the slope of the graph shows a remarkably decreasing trend after five or seven factors are extracted. In the case of the Scree test in Figure 5, if the slope is not clear, there is a possibility of subjective interpretation, so the number of factors of this tool was determined to be six by applying several criteria in combination.

### 3.2. Assessment of Factor Structure and Reliability 

The results of the factor loading for each factor were 0.79 to 0.89 for Factor 1, 0.76 to 0.88 for Factor 2, 0.76 to 0.83 for Factor 3, 0.73 to 0.84 for Factor 4, and 0.67 to 0.88 for Factor 5. Factor 6 was extracted as 0.68~0.85, and there were no items with factor loadings less than 0.30. The eigenvalues of each factor were verified as 5.66 for Factor 1, 5.01 for Factor 2, 5.00 for Factor 3, 4.93 for Factor 4, 4.71 for Factor 5, and 4.58 for Factor 6. Moreover, the explanatory power of each factor was 15.73% for Factor 1, 13.92% for Factor 2, 13.90% for Factor 3, 13.71% for Factor 4, 13.08% for Factor 5, and 12.72% for Factor 6, so all six factors contributed to the total variance of 83.06% that was explained (Table 11).

Due to the exploratory nature of developing measurement scales, the authors checked for scale reliability in addition to the peer group evaluation and pilot survey feedback to ensure the quality of the instrument and data. The pool of 36 items was factor-analyzed to verify its dimensionality. As Churchill [121] recommended, the process began by calculating the coefficient alpha values for each of the six sets of items, as shown in Table 6 and Table 7. These six dimensions were then subjected to varimax rotation. Table A2 presents the rotated factor loadings for 36 structured items as well as the alpha values for the six dimensions obtained from the OI attitude assessment survey data set. As anticipated, the result indicates that the questionnaire components are distinctively grouped into six adequate factors; the cumulative percentage marks 83.06%, which is much higher than the normally acceptable level of 60.0%. The coefficient alpha values ranged from 0.912 to 0.978 across the six dimensions. Table 11 also shows the coefficient alpha values for the six COIAA dimensions. Item commonalities are considered high if they are greater than 0.80 [117], but the standard practice in social science is to employ low to moderate commonalities of 0.40 to 0.70. On the table, the factor loading cutoff criterion of 0.30 is chosen [122]. The results indicate that the six-factor structure of the COIAA scale collectively had a clean factor structure and individually exhibited reliability coefficients greater than the minimum recommended value of 0.70, indicating a high level of reliability for each questionnaire section.

Some observations about the contents and structure of the COIAA are worth mentioning. First, the COIAA contains proven measurement questions regarding OI attitude. Based on a thorough review of the literature, including published scholarly articles and academic papers, a multidimensional OI aspect of role perspectives and loci of activities are well aligned. Second, an organization’s attitude toward OI, in terms of validation of intention to act, is a fair predictor of open innovation behavior [123,124]. Furthermore, a questionnaire consisting of three components can be used as a baseline for an organization’s OI attitude [16]. Third, the factor-loading pattern for COIAA’s subscales confirms that intra- and inter-organizational divisions are well understood as they are currently structured.

### 3.3. Assessment of Validity 

While the high reliabilities and consistent factor structure of the COIAA’s six dimensions support the scale’s trait validity [125], high-reliability coefficients and consistent dimensionality are insufficient for establishing construct validity [121]. A content validity assessment must also be performed to ensure the COIAA scale’s construct validity. Its purpose is to examine “(a) the thoroughness with which the construct’s domain was established and (b) the adequacy of the scale items in representing all facets of the domain” [116]. As presented in an earlier section, the COIAA fulfills both criteria. The scale emerged from pretest studies assisted by business school students in graduate programs, colleagues in several companies, and academic advisors. Based on the results of the preliminary evaluation of the 36-item battery, a focus group interview was performed to ensure that the objectives of the questionnaire were straightforward and unambiguous. The interview results suggested that all questionnaire items were clearly understood and could be answered as intended. The next section provides a follow-up procedure and summarizes key insights pertaining to organizational open innovation attitude assessment.

*Confirmation of the Factor Structure.* In addition to content validity, the authors undertook an empirical evaluation of the COIAA scale’s construct validity. The second-order confirmatory factor analysis (CFA) employed AMOS 21 and maximum likelihood estimation to test the theoretical assumptions that (i) the items of the scale attitudes reflected on the six dimensions identified in the EFA (internal and external transfer, absorption, and brokerage) and (ii) the six dimensions (first-order constructs) reflected facets of an overall OI attitude (second-order construct).

Convergent validity was verified through estimated average variance extracted (AVE) and construct (or composite) reliability (CR). As demonstrated in Table 12, all composite reliabilities are 0.90 or higher, and AVEs are 0.650 or higher. Furthermore, discriminant validity was verified by comparing the square root of the AVE value with the correlation coefficient. First, the fitness index of the model was /df = 1.770 (≤2.0), Tucker–Lewis index (TLI) = 0.924 (≥0.9), comparative fit index (CFI) = 0.932 (≥0.9), and root mean square error of approximation (RMSEA) = 0.076 (≤0.08), all of which were evaluated as excellent [126,127]. The estimate for factor loading should be 0.70 or higher, and the t-value for the significance of the estimated loading should be 1.965 or higher to verify the convergent validity. Additionally, construct reliability should be 0.70 or higher, and the AVE value should be 0.05 or higher [128,129]. As shown in Table 12, all previously mentioned measurement items met the criteria.

Meanwhile, to satisfy the discriminant validity, the square root of the AVE must be greater than the absolute value of the correlation coefficient of each variable. As shown in Table 13, the absolute value of the correlation coefficient was consistently smaller than the AVE value, so it was determined that the discriminant validity of the scale proposed in this study was satisfied.

**Figure 6:**
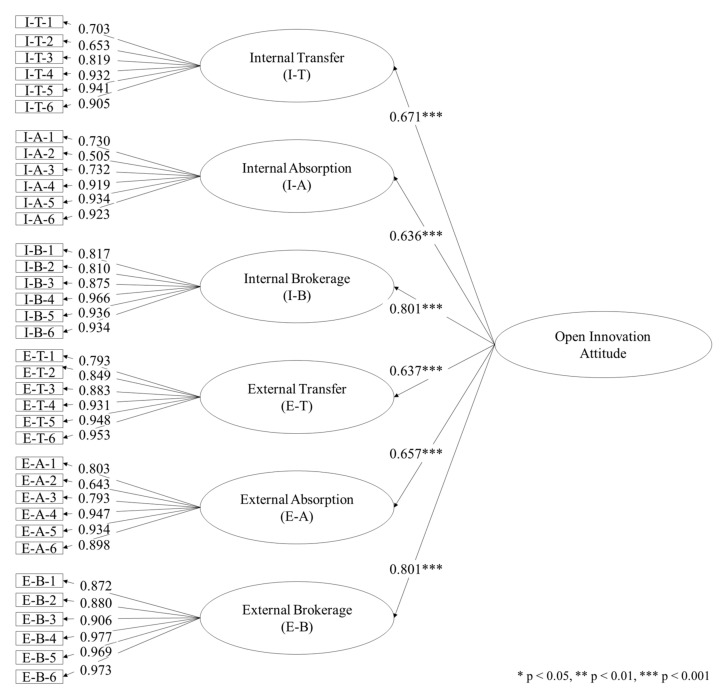
Second-order CFA with six dimensions as first-order construct and OI attitude as second-order construct.

Figure 6 displays the factor loadings of each item and squared multiple correlations of the six first-order factors. The squared multiple correlations of the six dimensions and the indicator reliabilities for each item exceed the recommended minimum of 0.4 [130]. Altogether, the findings confirm the theoretically assumed structure of the data and imply a high factorial validity of the scale. The model criteria for indices are /df = 1.828 (≤2.0), TLI = 0.918 (≥ 0.9), CFI = 0.926 (≥0.9), and RMSEA = 0.079 (≤0.08), all of which were evaluated as satisfactory (Byrne, 2001 [126]; Steiger, 1990 [127]).

## 4. Conclusions and Future Research Direction

Contributing to the growing literature on organizational attitude measurement in an OI context, this paper identifies the organizational attitudes necessary for executing OI and areas that require further development regarding role perspectives and loci of OI activities. This research contributes to the theory and practice aspects of a firm’s OI strategy by developing an initial OI attitude assessment instrument at the organizational level and presenting original empirical analysis results and development of the COIAA. Through rigorous literature review and empirical analysis with an adequate research model, the authors explored the behavioral aspects of the firm toward OI. This research’s contributions can be summarized as follows.

### 4.1. Theoretical Contributions

As Bogers et al. [49] recognized, OI is a developing concept that still lacks theoretical grounding, widely accepted measurement instruments, and quantitative empirical studies. This research contributes to theory building by proposing and validating measurement scales for OI attitude at the organization level. This COIAA tool is generic and can be used for OI readiness analysis in firms that reflect aspects of the tripartite role perspectives and loci of OI activities.

By applying this validated assessment survey questionnaire, constructs related to OI activities and the corporate environment were validated and analyzed (see Table 11, Table 12 and Table 13). The proposed COIAA builds upon the existing literature [35,49,52,57,72] while achieving more than the conceptual, case-based empirical insights of previous research by proposing a generally applicable integrated method for assessing critical OI attitudes across all organizational settings. Sequentially, the measurement tool facilitates the construction of a 2 × 3 assessment framework and links organizational activities to particular organizational roles in the OI ecosystem. The core of the assessment scales is a distinct OI attitude, supported by intra-organizational and inter-organizational attitudes, thus reinforcing the conceptual links between the firm’s role perspectives and the loci of OI activities. The validated empirical data indicate that, rather than focusing separately on internal/external organizational activities or role perspectives, such as transfer, absorption, and brokerage, many firms view OI as a holistic process and treat the organization as part of an OI ecosystem involving collaboration at different levels. Moreover, this framework responds to recent demands for a more integrated approach to the different areas and levels of analysis in OI studies [49,131].

### 4.2. Practical Implications

The results of this research build links between perceived organizational OI attitudes according to role perspectives and areas of improvement for OI performance (see Table 11, Table 12 and Table 13). This research also provides insights for stakeholders of different organizational units and functions by demonstrating how collective individual OI attitude status can strengthen the potential of intentions to act upon OI activities at the organizational level by articulating categories of the requisite behavioral reflections for executing OI. Additionally, it offers insights into an organization’s strategic direction regarding collaborative activities and the construction of organizational competencies for OI [49].

In practical settings, the following implications support the identification and development areas of OI in organizations. Based on the COIAA framework, managers can trace a firm’s OI attitude, which derives from perceived current status, to facilitate OI activities in the organization. This framework includes specific knowledge or information regarding the industrial market ecosystem, taking a holistic approach to firms’ roles in transfer, absorption, and brokerage. Additionally, the assessment scale can be applied in both intra- and inter-organizational settings. The overview of a firm’s role perspectives and loci of OI activities in the industry ecosystem provides a contribution to the discussion about how firms can evaluate and design an OI roadmap by recognizing the current environmental status that enables the identification of areas of development or strategic focus.

To date, the brokerage role in an OI ecosystem has been somewhat neglected, and the study results support the meaningful functional role of a knowledge and technology broker, also viewed as an intermediary, mediator, or liaison depending on context. The multidimensional assessment of intra- and inter-organizational OI activities status can help firms recognize a unique competitive advantage or other areas of improvement in the OI ecosystem, as demonstrated in both reliability and validity test results for the role of brokerage (see Table 11, Table 12 and Table 13). Ultimately, the COIAA framework introduced in this study provides a general assessment methodology for cross-industrial organizations. The authors firmly believe that a successful assessment of organizational OI attitude will provide a clearer understanding of a firm’s general capabilities including its corporate environment, management directions, and the promotion of its OI culture.

It is also important to note that although OI attitude measurement variables were extracted for assessment purposes, firms can use the questionnaire in a practical setting for various purposes such as stimulating an organization’s OI activities motivation and tracking stakeholder awareness trends regarding in/external OI activities status regularly. Ultimately, results from these and related data sources can provide meaningful insights into topics such as the extent to which the development aspects of the OI role perspective should be prioritized, which areas of in/external OI activities or processes should be enhanced, and which types of support are required to assist stakeholders experiencing obstacles in executing OI activities.

### 4.3. Limitations and Future Research Directions

The findings from the COIAA framework provided insights about the structure of an organization’s OI attitude constructs and validated a scale to measure them. This study has also stimulated the enhancement and empirical validation of models in which COIAA is a core construct. For instance, frameworks embracing various criteria (e.g., demographics, socio-economics) and consequences (e.g., OI performance results) of overall OI attitudes are worthy of investigation [132]. Exploring more detailed models (e.g., brokerage-specific OI activities and performance regarding the in/external scope of an organization) is another potentially rewarding theme for research.

There are also areas for improvement in comparative studies of COIAA across different corporate environments and cultures. Do the patterns of COIAA-related findings for multi-national firms operating in South Korea hold in other countries, considering the nuances of a questionnaire interpreted by a society’s subjective norms and collective or individual nature? Would segmenting the in/external organizational sphere within an internationally operating corporation require alternative aspects of measurement constructs? Empirical study-based outcomes can contribute to strengthening internationally proven organizational OI theory and practice. Although the sample size was sufficient for the analysis performed, it would be helpful to collect a broader range of samples from more firms and respondents as well as cross-border business operations. By assuming cultural variations, an interesting research direction could be realized by extracting variables measuring cultural characteristics in the future. Another potential avenue for research is tracking COIAA within and across different organizations over time to find possible reasons for any changes or lack thereof.

In addition to OI attitude assessment, scale development in corporate OI capability assessment is also an invaluable research topic. Building on an organization’s attitude toward OI discussed in this paper by measuring the capabilities of each organization to perform OI activities, such as possessing competent human resources, cooperation networks, work processes, infrastructure, corporate culture, and leadership, would help identify an organization’s needs in each OI capability categories and allow for the establishment of an improvement plan to enhance such capabilities.

In conclusion, this research makes a significant contribution to the study of OI by proposing measurement scales for an organization’s OI attitude as reflected in its in/external scope of activities and role perspectives in the OI ecosystem and by developing the COIAA framework. This research invites discussion of required and desired organizational culture and processes in firms implementing OI. It also proposes an interdisciplinary approach by seeking to integrate OI and psychometric measurement scales into the development of research streams, which can contribute to the development of practices related to a firm’s OI strategy management, including the selection of focus areas in terms of roles and loci in the OI environment, internal organizational training, reward systems, work process management, and an understanding of the role of OI stakeholders. The questionnaire and validated scales also provide a unique tool for evaluating current and desired OI activities-related attitudes. The authors believe that the findings of this study will be of great value to a broad range of OI academics and industry professionals and will stimulate constructive debate around the framework and measurement constructs for OI as well as organizational capabilities related to specific OI needs. Such debates will not only deepen understanding of the construct but also offer practical solutions to meet the increased demand for sound evaluation methods for successful outcomes in the OI ecosystem.

## Figures and Tables

**Table 1:** Indicative organizational OI capabilities from the literature.

Organizational Capability	Description of Capabilities	Author(s) (Year)
Market Sensing	Leverage the discoveries of others. Explore many external sources of innovation.	Dahlander and Gann [22]
	Create a synergy between own processes and externally available ideas.	Herzog [23]
Network and Process	Improve and develop systematic processes to enable inter-departmental cooperation. Create well-defined routines to build and define the OI tasks.	Sivam et al. [4]
	Prioritize innovation process based on purposively managed knowledge flows across boundaries of organization, using (non) pecuniary mechanisms aligned with the business model.	Chesbrough and Bogers [24]
	Emulate the company’s innovation paradigm. Transform solid boundaries into a more semi-permeable layer to enable innovation to move more easily between the company’s external environment and internal innovation process.	Gassmann and Enkel [25]
Corporate Culture	Explain trait or characteristic. Support product/process innovation.	Yun et al. [26] Lee et al. [27]
	Balance innovation and daily tasks, communication problems, aligning partners, organization of innovation.	Van de Vrande et al. [28]
	Demonstrate organizer’s appreciation. Demonstrate peers’ appreciation.	Leimeister et al. [29]
Reward Policy	Demonstrate organizer’s appreciation. Demonstrate peers’ appreciation.	Leimeister et al. [29]
	Follow organizational principles, convey management’s conviction that employee involvement is desirable.	Van de Vrande et al. [30]
	Involve employees in the innovation process to increase their motivation and commitment.	
Organizational Leadership	Provide extrinsic incentives, such as financial rewards, in addition to intrinsic incentives, such as appreciation to people who push, modify, or drop the innovation.	Herzog [23]
	Offer employees’ education or training. Create awareness among employees about how open innovation can further advance their creativity and innovativeness. Offer incentives, monetary and non-monetary, to employees to encourage them to embrace open innovation.	Barham et al. [31]
	Encourage management to bring in cultural change, new thinking, and clear mandates to access external innovation.	Slowinski et al. [32]
	Provide top-down direction and encouragement for OI practices.	Chesbrough and Crowther [33]

**Table 2:** Classification of role perspective in OI ecosystem.

Role Perspective	Examples of OI Activities	Author (Year)
Transfer (Outbound, Inside-out, Boundary-spanning)	Support venturing, licensing IP to other firms, participation in other firms.	Van de Vrande et al. [28]
	Allow sharing of the knowledge, costs, and risks of uncertain innovative projects.	Bogers et al. [49]
	Recognize that licensing positively affects a company’s entrepreneurial learning and contextual status.	Hu et al. [54]
	Enhance technological knowledge transfer.	Gassmann and Enkel [25]; Lichtenthaler [55];
	Commercialize external technology.	Kutvonen [56]; Lichtenthaler and Ernst [57]; Lichtenthaler [58]
	Encourage out-licensing.	Lichtenthaler and Ernst [57]
	Consider selling and out-licensing an effective strategy in commercialization.	Kollmer and Dowling [59]
	Encourage spin-off.	Chesbrough [35]; Chesbrough and Rosenbloom [60]
	Allow the use of different sources for innovation projects through division of labor.	Schilling [61]
Absorption (Inbound, Outside-in, Spin-in)	Encourage customer involvement, outsourcing R&D.	Van de Vrande et al. [28]
	Encourage networking.	Van de Vrande et al. [28]; Salavisa et al. [62]
	Emphasize cooperation.	Mention and Asikainen [63]; Trigo and Vence [64]; Mention [65]; Tether [66]
	Support coopetition.	Mention [65]
	Encourage institutional collaboration.	Aschhoff and Schmidt [67]; Belderbos et al. [68]
	Purchase scientific services.	Chiaroni et al. [69]
	Facilitate in-licensing from external technology source.	Tsai and Wang [70]
	Facilitate a capability-based framework for open innovation, supplementing absorptive capacity.	Lichtenthaler and Lichtenthaler [40]; Lichtenthaler [71]
	Emphasize spin-in or outside-in knowledge across organizational boundaries as a core process.	Gassmann (2006) [72]
	Use open, wide, and deep innovation strategy to improve company’s external search for knowledge.	Laursen and Salter [73]
	Focus on acquisition.	Vanhaverbeke et al. [74]
Brokerage (Intermediation, Mediation)	Enhance inter-organizational innovation collaboration.	Enkel et al. [36]; Huston and Sakkab [53]
	Integrate a diverse set of innovation actors in the OI ecosystem.	West and Bogers [37]
	Represent a large knowledge source for firms’ OI.	Agogué et al. [48]; Laursen and Salter [73]
	Reduce internal fear of experimentation. Motivate external stakeholders’ contributions. Support connectivity between diverse actors. Facilitate entry and collaboration of various new actors.	Bogers et al. [49]; Moellers et al. [75]
	Provide external knowledge for OI strategies.	Huggins and Prokop [50]
	Broker technology patents in the market.	Benassi and Di Minin [51]
	Manage the knowledge path between public and private parties of the innovation system.	Chesbrough [76]
	Support clusters to enhance OI in particular industries.	Santos [77]
	Represent a large knowledge source for firms’ OI.	Piller and West [78]
	Enhance the performance of ecosystem members in competitive markets.	Lopez-Berzosa and Gawer [79]
	Participate in firms’ OI processes by communicating their needs and preferences.	Bogers et al. [80]
	Support collaboration and joint research programs.	Dittrich and Duysters [81]
	Create network brokerage of collaborative relationships.	Fleming and Waguespack [82]
Exploration	Search firm’s technological competency and problem boundaries. Invent and absorb knowledge in internal and external firm settings. Acquire knowledge from external sources. Generate new knowledge inside the firm.	Brunswicker et al. [38] Lichtenthaler and Lichtenthaler [40] Lane et al. [83] Smith et al. [84]
Exploitation	Transform and connect knowledge in internal and external firm settings. Convert knowledge from internal and external sources into new products. Replicate new approaches in diverse context.	Lichtenthaler and Lichtenthaler [40] Moore [41] Zollo and Winter [85]
Ambidexterity	Align internal and external knowledge management processes. Achieve alignment and adaptability simultaneously at a business-unit level.	Gibson and Birkinshaw [39] Lichtenthaler and Lichtenthaler [40]

**Table 3:** Examples of OI activities by locus.

Locus of OI Activity	Examples of OI Activities	Author (Year)
	Outbound licensing of intellectual property.	Chesbrough and Crowther [33]
	Manage intellectual property.	Chesbrough [35]
	Rethink managerial and governance structures that motivate participants. Utilize external knowledge in the firm’s organizational innovation outputs.	Bogers et al. [49]
	Stimulate corporate entrepreneurship.	Chesbrough et al. [52]
	Align incentives for better external use of ideas.	Chesbrough [86]
	Transform ideas into commercial outputs. Facilitate collaboration by building internal cross-unit networks. Shape high-quality links within and across units and individuals. Transform market needs into technology briefs.	Hansen and Brikinshaw [87]
	Increase the firm’s absorptive capacity.	Bogers and Lhuillery [90]
	Help firm obtain unique dynamic capabilities.	Teece [91]
	Build firm’s strategic alliances with new technology supporters.	Rothaermel and Hess [92]
	Provide individuals and groups with toolkits for user innovation.	Piller and Walcher [93]
	
	Maximize internal innovation returns.	West and Gallagher [94]
	Help firm to gain benefit from external research projects.	Colyvas et al. [95]
External Scope of Activities (Inter-organizational)	Enhance inter-organizational innovation collaboration.	Enkel et al. [36]
	Facilitate the establishment of boundary-spanning innovation activities.	Chesbrough et al. [52]
	Help firm obtain external paths.	Biemans [96]
	Relate R&D unit activities to innovation industry. Acquire new business partners to commercialize new products and ideas.	Martinez-Conesa et al. [97]
	Exploit technology externally.	Dodgson et al. [98]
	Maintain connections among OI external sources and relevant network partners.	De Jong and Hulsink [99]
National Scope of Activities	Provide a proper breeding ground for the development and effectiveness of relational governance mechanisms. Collaborate resources among firms in the same region.	Clauss and Spieth [89] Huggins and Johnston [100]
International Scope of Activities	Cooperate with innovation partners in international network scope.Internalize corporate enterprises and innovation schemes. Establish international joint ventures to ensure survival, increase competitiveness, and enter new markets. Collaborate OI networks across international borders.	Clauss and Spieth [89] Herstad [101] Mohr and Puck [102] De Meyer [103]

**Table 4:** Hollis reliability test for Delphi Survey (1).

Categories		Expert A–Expert B	Expert A–Expert C	Expert B–Expert C
OI Locus of Activities	Items consistency	1	1	1
	Items inconsistency	1	1	1
	Total	2	2	2
	Coefficient	0.5000	0.5000	0.5000
OI Role Perspectives	Items consistency	1	1	2
	Items inconsistency	2	2	1
	Total	3	3	3
	Coefficient	0.3333	0.3333	0.3333
Total	Items consistency	2	2	3
	Items inconsistency	3	3	2
	Total	5	5	5
	Coefficient	0.4000	0.4000	0.6000

Note: Expert A: Business; B: Public; C: Academic.

**Table 5:** Hollis reliability test for Delphi Survey (2).

Categories		Expert A–Expert B	Expert A–Expert C	Expert B–Expert C
OI Locus of Activities	Items consistency	2	1	1
	Items inconsistency	0	1	1
	Total	2	2	2
	Coefficient	1.0000	0.5000	0.5000
OI Role Perspectives	Items consistency	2	2	3
	Items inconsistency	1	1	0
	Total	3	3	3
	Coefficient	0.6667	0.6667	1.0000
Total	Items consistency	4	3	4
	Items inconsistency	1	2	1
	Total	5	5	5
	Coefficient	0.8000	0.6000	0.8000

Note: Expert A: Business; B: Public; C: Academic.

**Table 6:** Item analysis for COIAA pilot survey (1) (N = 15).

Items	M ± SD	Skewness	Kurtosis	Corrected Item- Total Correlation	Cronbach’s α if Item is Deleted
IT01	5.00 ± 1.51	−0.55	−0.58	0.48	0.97
IT02	4.88 ± 1.51	−0.72	−0.14	0.48	0.97
IT03	5.12 ± 1.15	−0.81	−0.05	0.52	0.97
IT04	5.08 ± 1.65	−0.64	−0.60	0.59	0.97
IT05	4.94 ± 1.61	−0.72	−0.25	0.64	0.97
IT06	5.04 ± 1.63	−0.65	−0.45	0.62	0.97
IT07	4.86 ± 1.23	−0.56	0.35	0.53	0.97
IT08	4.48 ± 1.45	−0.50	−0.41	0.52	0.97
IT09	4.13 ± 1.48	−0.44	−0.80	0.64	0.97
IT10	4.06 ± 1.53	−0.22	−0.72	0.57	0.97
IA01	5.53 ± 1.30	−0.87	0.49	0.54	0.97
IA02	5.58 ± 1.06	−0.63	0.30	0.51	0.97
IA03	5.42 ± 1.35	−0.73	−0.05	0.49	0.97
IA04	5.79 ± 1.15	−0.87	0.27	0.40	0.97
IA05	5.70 ± 1.13	−0.91	1.16	0.47	0.97
IA06	5.67 ± 1.12	−0.96	1.36	0.51	0.97
IA07	4.08 ± 1.65	−0.17	−0.80	0.55	0.97
IA08	3.79 ± 1.66	−0.14	−1.05	0.59	0.97
IA09	3.67 ± 1.57	−0.10	−0.94	0.62	0.97
IA10	3.79 ± 1.53	−0.22	−0.73	0.44	0.97
IB01	4.87 ± 1.52	−0.33	−0.51	0.64	0.97
IB02	4.75 ± 1.47	−0.45	−0.16	0.69	0.97
IB03	4.75 ± 1.70	−0.45	−0.49	0.64	0.97
IB04	4.85 ± 1.59	−0.48	−0.38	0.66	0.97
IB05	4.83 ± 1.63	−0.50	−0.48	0.70	0.97
IB06	4.93 ± 1.59	−0.56	−0.27	0.68	0.97
IB07	4.88 ± 1.30	−0.85	0.68	0.35	0.97
IB08	4.40 ± 1.50	−0.45	−0.46	0.51	0.97
IB09	4.29 ± 1.48	−0.40	−0.55	0.53	0.97
IB10	3.79 ± 1.49	−0.21	−0.77	0.56	0.97

(1) *** *p* < 0.01, ** *p* < 0.05, * *p* < 0.010; (2) excluded items due to lower corrected item-total correlation, and excluded items due to Cronbach’s alpha if item is deleted.

**Table 7:** Item analysis for COIAA pilot survey (2) (N = 15).

Items	M ± SD	Skewness	Kurtosis	Corrected Item- Total Correlation	Cronbach’s α if Item is Deleted
ET01	3.70 ± 1.46	0.49	−0.60	0.59	0.97
ET02	3.57 ± 1.58	0.12	−0.70	0.58	0.97
ET03	3.64 ± 1.46	0.15	−0.59	0.60	0.97
ET04	3.93 ± 1.54	−0.23	−0.51	0.62	0.97
ET05	3.98 ± 1.54	−0.21	−0.48	0.61	0.97
ET06	4.07 ± 1.51	−0.22	−0.48	0.65	0.97
ET07	4.54 ± 1.47	−0.67	0.08	0.63	0.97
ET08	4.50 ± 1.57	−0.34	−0.53	0.61	0.97
ET09	3.90 ± 1.56	−0.18	−0.80	0.69	0.97
ET10	3.66 ± 1.53	−0.36	−0.76	0.55	0.97
EA01	5.29 ± 1.20	−0.78	1.13	0.49	0.97
EA02	4.87 ± 1.29	−0.45	−0.02	0.54	0.97
EA03	5.01 ± 1.35	−0.69	0.56	0.54	0.97
EA04	5.32 ± 1.26	−0.89	1.40	0.39	0.97
EA05	5.35 ± 1.21	−0.62	0.63	0.43	0.97
EA06	5.32 ± 1.26	−0.78	0.72	0.44	0.97
EA07	3.61 ± 1.72	0.18	−1.03	0.57	0.97
EA08	3.65 ± 1.70	−0.04	−1.17	0.58	0.97
EA09	3.55 ± 1.69	−0.01	−1.07	0.66	0.97
EA10	3.66 ± 1.64	−0.13	−1.03	0.60	0.97
EB01	4.29 ± 1.63	−0.31	−0.65	0.71	0.97
EB02	4.14 ± 1.63	−0.22	−0.68	0.74	0.97
EB03	4.18 ± 1.76	−0.18	−0.90	0.76	0.97
EB04	4.31 ± 1.72	−0.38	−0.73	0.73	0.97
EB05	4.41 ± 1.71	−0.49	−0.54	0.71	0.97
EB06	4.40 ± 1.73	−0.50	−0.56	0.73	0.97
EB07	4.14 ± 1.72	−0.42	−0.95	0.65	0.97
EB08	4.05 ± 1.68	−0.36	−0.82	0.61	0.97
EB09	3.90 ± 1.70	−0.23	−0.89	0.67	0.97
EB10	3.58 ± 1.66	−0.09	−1.11	0.61	0.97

(1) *** *p* < 0.01, ** *p* < 0.05, * *p* < 0.10; (2) excluded items due to lower corrected item-total correlation, and excluded items due to Cronbach’s alpha if item is deleted.

**Table 8:** Item analysis for COIAA main survey (N = 134).

Items	M ± SD	Skewness	Kurtosis	Corrected Item- Total Correlation	Cronbach’s α if Item is Deleted
IT01	5.00 ± 1.51	−0.55	−0.58	0.54	0.74
IT02	4.88 ± 1.51	−0.72	−0.14	0.50	0.75
IT03	5.12 ± 1.53	−0.81	−0.05	0.56	0.86
IT04	5.08 ± 1.65	−0.68	−0.60	0.66	0.89
IT05	4.94 ± 1.61	−0.72	−0.25	0.70	0.88
IT06	5.04 ± 1.63	−0.65	−0.45	0.67	0.82
IA01	5.53 ± 1.30	−0.87	0.49	0.64	0.74
IA02	5.58 ± 1.06	−0.63	0.30	0.59	0.82
IA03	5.42 ± 1.35	−0.73	−0.05	0.59	0.74
IA04	5.79 ± 1.15	−0.87	0.27	0.53	0.88
IA05	5.70 ± 1.13	−0.91	1.16	0.60	0.86
IA06	5.67 ± 1.12	−0.96	1.36	0.60	0.87
IB01	4.87 ± 1.52	−0.33	−0.51	0.71	0.80
IB02	4.75 ± 1.47	−0.45	−0.16	0.70	0.86
IB03	4.75 ± 1.70	−0.45	−0.49	0.69	0.87
IB04	4.85 ± 1.59	−0.48	−0.38	0.75	0.92
IB05	4.83 ± 1.63	−0.50	−0.48	0.76	0.92
IB06	4.93 ± 1.59	−0.56	−0.27	0.75	0.93
ET01	3.70 ± 1.46	0.04	−0.60	0.61	0.82
ET02	3.57 ± 1.58	0.12	−0.70	0.59	0.89
ET03	3.64 ± 1.46	0.15	−0.59	0.62	0.88
ET04	3.93 ± 1.54	−0.23	−0.51	0.64	0.89
ET05	3.98 ± 1.47	−0.21	−0.48	0.67	0.90
ET06	4.07 ± 1.51	−0.22	−0.48	0.69	0.89
EA01	5.29 ± 1.20	−0.78	1.13	0.61	0.78
EA02	4.87 ± 1.29	−0.45	−0.02	0.59	0.75
EA03	5.01 ± 1.35	−0.69	0.56	0.66	0.84
EA04	5.32 ± 1.26	−0.89	1.40	0.56	0.91
EA05	5.35 ± 1.21	−0.62	0.63	0.59	0.88
EA06	5.32 ± 1.26	−0.78	0.72	0.58	0.87
EB01	4.29 ± 1.63	−0.31	−0.65	0.76	0.89
EB02	4.14 ± 1.63	−0.22	−0.68	0.77	0.92
EB03	4.18 ± 1.76	−0.18	−0.90	0.79	0.90
EB04	4.31 ± 1.72	−0.38	−0.73	0.77	0.95
EB05	4.41 ± 1.71	−0.49	−0.54	0.78	0.94
EB06	4.40 ± 1.73	−0.50	−0.56	0.77	0.94

(1) *** *p* < 0.01, ** *p* < 0.05, * *p* < 0.10; (2) excluded items due to lower corrected item-total correlation and excluded items due to Cronbach’s alpha if item is deleted.

**Table 9:** Descriptive sample statistics.

Characteristics	Respondents	Sample Size (n)	Proportion (%)
Gender	Male	106	79.1%
	Female	28	20.9%
Age	20 s	3	2.2%
	30 s	68	50.7%
	40 s	59	44.0%
	Over 50	4	3.0%
Business Activity Area	IT/Software	16	11.9%
	Sales/Marketing	37	27.6%
	HR/Education	9	6.7%
	Strategy/Planning	49	36.6%
	Finance/Accounting	10	7.5%
	Others	13	9.7%

**Table 10:** KMO and Bartlett’s test.

Kaiser–Meyer–Olkin Measure of Sampling Adequacy		0.916
Bartlett’s Test of Sphericity	Approx. Chi-Square	6308.071
	Df	630
	Sig.	0.000

**Table 11:** Exploratory factor analysis for COIAA main survey (N = 134).

Items	Communality	Components					
		1	2	3	4	5	6
IT01	0.71	0.12	0.77	0.16	0.07	0.25	0.03
IT02	0.66	0.13	0.77	0.14	0.02	0.14	0.08
IT03	0.86	0.09	0.88	0.17	0.05	0.19	0.05
IT04	0.84	0.11	0.82	0.23	0.13	0.24	0.13
IT05	0.83	0.18	0.79	0.16	0.26	0.23	0.13
IT06	0.78	0.19	0.76	0.24	0.16	0.24	0.09
IA01	0.69	0.09	0.23	0.32	0.16	0.67	0.21
IA02	0.78	0.08	0.34	0.15	0.07	0.77	0.17
IA03	0.69	0.15	0.42	0.20	-0.01	0.64	0.18
IA04	0.89	0.07	0.20	0.07	0.11	0.88	0.23
IA05	0.85	0.08	0.21	0.14	0.10	0.83	0.26
IA06	0.85	0.01	0.21	0.20	0.13	0.83	0.23
IB01	0.79	0.24	0.14	0.76	0.25	0.20	0.12
IB02	0.81	0.23	0.29	0.78	0.17	0.12	0.11
IB03	0.85	0.16	0.23	0.83	0.19	0.12	0.16
IB04	0.91	0.11	0.18	0.83	0.29	0.21	0.21
IB05	0.86	0.14	0.24	0.76	0.36	0.21	0.12
IB06	0.84	0.13	0.25	0.76	0.31	0.21	0.16
ET01	0.77	0.79	0.14	0.13	0.27	0.14	0.12
ET02	0.86	0.87	0.18	0.09	0.19	-0.03	0.13
ET03	0.89	0.89	0.18	0.13	0.12	0.01	0.15
ET04	0.86	0.86	0.15	0.14	0.22	0.08	0.11
ET05	0.86	0.84	0.06	0.16	0.25	0.17	0.17
ET06	0.86	0.83	0.09	0.20	0.27	0.14	0.12
EA01	0.77	0.14	0.14	0.15	0.18	0.22	0.79
EA02	0.68	0.31	0.21	0.09	0.24	-0.08	0.68
EA03	0.80	0.31	0.15	0.23	0.12	0.12	0.77
EA04	0.89	0.08	-0.14	0.14	0.13	0.34	0.85
EA05	0.84	0.05	0.03	0.15	0.20	0.32	0.81
EA06	0.76	0.09	0.08	0.05	0.24	0.34	0.74
EB01	0.86	0.40	0.10	0.28	0.73	0.10	0.22
EB02	0.88	0.42	0.17	0.28	0.74	0.02	0.21
EB03	0.89	0.37	0.13	0.31	0.75	0.11	0.23
EB04	0.95	0.26	0.l1	0.28	0.84	0.13	0.23
EB05	0.93	0.28	0.12	0.27	0.82	0.17	0.23
EB06	0.93	0.23	0.14	0.30	0.83	0.14	0.22
Eigenvalue		5.66	5.01	5.00	4.93	4.71	4.58
Explained variance (%)		15.73	13.92	13.90	13.71	13.08	12.72
Cumulative explained variance (%)		15.73	29.65	43,55	57.26	70.34	83.06
Kaiser–Meyer–Olkin test = 0.918; Bartlett’s test of sphericity: χ^2^ = 6370.374 (*p* < 0.001)							

(1) *** *p* < 0.01, ** *p* < 0.05, * *p* < 0.10.

**Table 12:** Results of convergent validity test.

Locus of OI Activity	Role Perspectives	Component	Estimate	S.E.	t-Value	CR	AVE
Internal	Transfer	I-T-1	1.000			0.931	0.695
		I-T-2	0.924	0.09	10.463 ***		
		I-T-3	1.142	0.09	12.091 ***		
		I-T-4	1.441	0.14	10.416 ***		
		I-T-5	1.410	0.14	10.472 ***		
		I-T-6	1.380	0.14	10.147 ***		
	Absorption	I-A-1	1.000			0.915	0.650
		I-A-2	0.753	0.11	7.068 ***		
		I-A-3	1.075	0.12	8.971 ***		
		I-A-4	1.120	0.10	11.391 ***		
		I-A-5	1.126	0.10	11.618 ***		
		I-A-6	1.102	0.10	11.452 ***		
	Brokerage	I-B-1	1.000			0.959	0.795
		I-B-2	0.952	0.07	14.022 ***		
		I-B-3	1.185	0.09	12.696 ***		
		I-B-4	1.239	0.08	14.862 ***		
		I-B-5	1.231	0.09	14.114 ***		
		I-B-6	1.196	0.09	14.005 ***		
External	Transfer	E-T-1	1.000			0.960	0.802
		E-T-2	1.129	0.07	16.652 ***		
		E-T-3	1.116	0.09	12.182 ***		
		E-T-4	1.237	0.09	13.189 ***		
		E-T-5	1.199	0.09	13.527 ***		
		E-T-6	1.243	0.09	13.656 ***		
	Absorption	E-A-1	1.000			0.935	0.709
		E-A-2	0.796	0.10	8.395 ***		
		E-A-3	1.090	0.11	10.424 ***		
		E-A-4	1.268	0.09	13.801 ***		
		E-A-5	1.207	0.09	13.463 ***		
		E-A-6	1.209	0.09	12.63 ***		
	Brokerage	E-B-1	1.000			0.975	0.867
		E-B-2	1.012	0.04	23.323 ***		
		E-B-3	1.127	0.05	22.194 ***		
		E-B-4	1.187	0.06	19.024 ***		
		E-B-5	1.169	0.06	18.552 ***		
		E-B-6	1.181	0.06	18.741 ***		

*** correlation is significant at *p* < 0.001.

**Table 13:** Results of discriminant validity test.

		Internal			External		
		Transfer	Absorption	Brokerage	Transfer	Absorption	Brokerage
Internal	Transfer	0.834					
	Absorption	0.623 **	0.806				
	Brokerage	0.549 **	0.542 **	0.892			
External	Transfer	0.392 **	0.289 **	0.460 **	0.895		
	Absorption	0.365 **	0.553 **	0.472 **	0.434 **	0.842	
	Brokerage	0.417 **	0.407 **	0.668 **	0.634 **	0.563 **	0.931

The diagonal is the square root of the mean variance extraction index, and the non-diagonal is the correlation between variables. * Correlation is significant at *p* < 0.05, ** correlation is significant at *p* < 0.01.

## Data Availability

The data presented in this study are available on request from the corresponding author. The data are not publicly available due to privacy protection for respondents.

## Appendix A

**Table A1:** Corporate Open Innovation Attitude Assessment (COIAA) Questionnaire.

Internal (Intra-Company) OI Attitude	*S. Disagree* *S. Agree*
I-1. Perceived Transfer-out Attitude	
*Members of **my team** (a) ______ work-related skills or knowledge **possessed by my team** (b)______*	
(1) (a) are interested in providing / (b) to other teams.	① ② ③ ④ ⑤ ⑥ ⑦
(2) (a) naturally provide / (b) to other teams.	① ② ③ ④ ⑤ ⑥ ⑦
(3) (a) feel that we should provide / (b) to other teams.	① ② ③ ④ ⑤ ⑥ ⑦
(4) (a) think providing / (b) to other teams will help my team.	① ② ③ ④ ⑤ ⑥ ⑦
(5) (a) think providing / (b) to other teams will improve my team’s performance.	① ② ③ ④ ⑤ ⑥ ⑦
(6) (a) think providing / (b) to other teams will strengthen my team’s capabilities.	① ② ③ ④ ⑤ ⑥ ⑦
I-2. Perceived Absorptive Attitude	
*Members of **my team** (a)______ work-related skills or knowledge **possessed by other teams** (b)______*	
(1) (a) are interested in taking in / (b) n/a	① ② ③ ④ ⑤ ⑥ ⑦
(2) (a) naturally accept taking in / (b) n/a	① ② ③ ④ ⑤ ⑥ ⑦
(3) (a) feel that we should be taking in / (b) n/a	① ② ③ ④ ⑤ ⑥ ⑦
(4) (a) think taking in / (b) will help my team.	① ② ③ ④ ⑤ ⑥ ⑦
(5) (a) think taking in / (b) will improve my team’s performance.	① ② ③ ④ ⑤ ⑥ ⑦
(6) (a) think taking in / (b) will strengthen my team’s capabilities.	① ② ③ ④ ⑤ ⑥ ⑦
I-3. Perceived Brokerage Attitude	
*Members of **my team** (a)_____ work-related skills or knowledge **possessed by other teams** (b)______*	
(1) (a) are interested in connecting / (b) in between them.	① ② ③ ④ ⑤ ⑥ ⑦
(2) (a) naturally accept connecting / (b) in between them.	① ② ③ ④ ⑤ ⑥ ⑦
(3) (a) feel that we should connect / (b) in between them.	① ② ③ ④ ⑤ ⑥ ⑦
(4) (a) think connecting / (b) in between them will help my team.	① ② ③ ④ ⑤ ⑥ ⑦
(5) (a) think connecting / (b) in between them will improve my team’s performance.	① ② ③ ④ ⑤ ⑥ ⑦
(6) (a) think connecting / (b) in between them will strengthen my team’s capabilities.	① ② ③ ④ ⑤ ⑥ ⑦
**External (Inter-Company) OI Attitude**	***S. Disagree*** ***S. Agree***
E-1. Perceived Transfer-out Attitude	
*Members of **my company** (a)____ work-related skills or knowledge **possessed by my company** (b)_____*	
(1) (a) are interested in providing / (b) to business partners.	① ② ③ ④ ⑤ ⑥ ⑦
(2) (a) naturally provide / (b) to business partners.	① ② ③ ④ ⑤ ⑥ ⑦
(3) (a) feel that we should provide / (b) to business partners.	① ② ③ ④ ⑤ ⑥ ⑦
(4) (a) think providing / (b) to business partners will help my company.	① ② ③ ④ ⑤ ⑥ ⑦
(5) (a) think providing / (b) to business partners will improve my company’s performance.	① ② ③ ④ ⑤ ⑥ ⑦
(6) (a) think providing / (b) to business partners will strengthen my company’s capabilities.	① ② ③ ④ ⑤ ⑥ ⑦
E-2. Perceived Absorptive Attitude	
*Members of **my company** (a)___ work-related skills or knowledge **possessed by business partners** (b)___*	
(1) (a) are interested in taking in / (b) n/a	① ② ③ ④ ⑤ ⑥ ⑦
(2) (a) naturally accept taking in / (b) n/a	① ② ③ ④ ⑤ ⑥ ⑦
(3) (a) feel that we should be taking in / (b) n/a	① ② ③ ④ ⑤ ⑥ ⑦
(4) (a) think taking in / (b) will help my company.	① ② ③ ④ ⑤ ⑥ ⑦
(5) (a) think taking in / (b) will improve my company’s performance.	① ② ③ ④ ⑤ ⑥ ⑦
(6) (a) think taking in / (b) will strengthen my company’s capabilities.	① ② ③ ④ ⑤ ⑥ ⑦
E-3. Perceived Brokerage Attitude	
*Members of **my company** (a)___ work-related skills or knowledge **possessed by business partners** (b)___*	
(1) (a) are interested in connecting / (b) in between them.	① ② ③ ④ ⑤ ⑥ ⑦
(2) (a) naturally accept connecting / (b) in between them.	① ② ③ ④ ⑤ ⑥ ⑦
(3) (a) feel that we should connect / (b) in between them.	① ② ③ ④ ⑤ ⑥ ⑦
(4) (a) think connecting / (b) in between them will help my company.	① ② ③ ④ ⑤ ⑥ ⑦
(5) (a) think connecting / (b) in between them will improve my company’s performance.	① ② ③ ④ ⑤ ⑥ ⑦
(6) (a) think connecting / (b) in between them will strengthen my company’s capabilities.	① ② ③ ④ ⑤ ⑥ ⑦

**Table A2:** 36-Item Corporate Open Innovation Attitude Assessment (COIAA) Scale: Factor Loadings After Varimax Rotation Matrix ^a^.

Locus of OI Activity	Role Perspectives	Variable		Component					
				1	2	3	4	5	6
*Internal*	Transfer	I-T. Perceived Transfer-out Attitude *Members of **my team** (a)______ work-related skills or knowledge **possessed by my team** (b)_____*							
		I-T-1	(a) are interested in providing/(b) to other teams.	**0.77**	-	-	-	-	-
		I-T-2	(a) naturally provide/(b) to other teams.	**0.77**	-	-	-	-	-
		I-T-3	(a) feel that we should provide/(b) to other teams.	**0.88**	-	-	-	-	-
		I-T-4	(a) think providing/(b) to other teams will help my team.	**0.82**	-	-	-	-	-
		I-T-5	(a) think providing/(b) to other teams will improve my team’s performance.	**0.79**	-	-	-	-	-
		I-T-6	(a) think providing/(b) to other teams will strengthen my team’s capabilities.	**0.76**	-	-	-	-	-
	Absorption	I-A. Perceived Absorptive Attitude *Members of **my team** (a)_____ work-related skills or knowledge **possessed by other teams** (b)____*							
		I-A-1	(a) are interested in taking in/(b) n/a	-	**0.67**	0.32	-	-	-
		I-A-2	(a) naturally accept taking in/(b) n/a	0.34	**0.77**	-	-	-	-
		I-A-3	(a) feel that we should be taking in/(b) n/a	0.42	**0.64**	-	-	-	-
		I-A-4	(a) think taking in/(b) will help my team.	-	**0.88**	-	-	-	-
		I-A-5	(a) think taking in/(b) will improve my team’s performance.	-	**0.83**	-	-	-	-
		I-A-6	(a) think taking in/(b) will strengthen my team’s capabilities.	-	**0.83**	-	-	-	-
	Brokerage	I-B. Perceived Brokerage Attitude *Members of **my team** (a)_____ work-related skills or knowledge **possessed by other teams** (b)____*							
		I-B-1	(a) are interested in connecting/(b) in between them.	-	-	**0.76**	-	-	-
		I-B-2	(a) naturally accept connecting/(b) in between them.	-	-	**0.78**	-	-	-
		I-B-3	(a) feel that we should connect/(b) in between them.	-	-	**0.83**	-	-	-
		I-B-4	(a) think connecting/(b) in between them will help my team.	-	-	**0.83**	-	-	-
		I-B-5	(a) think connecting/(b) in between them will improve my team’s performance.	-	-	**0.76**	-	-	0.36
		I-B-6	(a) think connecting/(b) in between them will strengthen my team’s capabilities.	-	-	**0.76**	-	-	0.31
*External*	Transfer	E-T. Perceived Transfer-out Attitude *Members of **my company** (a)___ work-related skills or knowledge **possessed by my company** (b)____*							
		E-T-1	(a) are interested in providing/(b) to business partners.	-	-	-	**0.79**	-	-
		E-T-2	(a) naturally provide/(b) to business partners.	-	-	-	**0.87**	-	-
		E-T-3	(a) feel that we should provide/(b) to business partners.	-	-	-	**0.89**	-	-
		E-T-4	(a) think providing/(b) to business partners will help my company.	-	-	-	**0.86**	-	-
		E-T-5	(a) think providing/(b) to business partners will improve my company’s performance.	-	-	-	**0.84**	-	-
		E-T-6	(a) think providing/(b) to business partners will strengthen my company’s capabilities.	-	-	-	**0.83**	-	-
	Absorption	E-A. Perceived Absorptive Attitude *Members of **my company** (a)____ work-related skills or knowledge **possessed by business partners** (b)____*							
		E-A-1	(a) are interested in taking in/(b) n/a	-	-	-	-	**0.79**	-
		E-A-2	(a) naturally accept taking in/(b) n/a	-	-	-	0.31	**0.68**	-
		E-A-3	(a) feel that we should be taking in/(b) n/a	-	-	-	0.31	**0.77**	-
		E-A-4	(a) think taking in/(b) will help my company.	-	0.34	-	-	**0.85**	-
		E-A-5	(a) think taking in/(b) will improve my company’s performance.	-	0.32	-	-	**0.81**	-
		E-A-6	(a) think taking in/(b) will strengthen my company’s capabilities.	-	0.34	-	-	**0.74**	-
	Brokerage	E-B. Perceived Brokerage Attitude *Members of **my company** (a)____ work-related skills or knowledge **possessed by business partners** (b)____*							
		E-B-1	(a) are interested in connecting/(b) in between them.	-	-	-	0.40	-	**0.73**
		E-B-2	(a) naturally accept connecting/(b) in between them.	-	-	-	0.42	-	**0.74**
		E-B-3	(a) feel that we should connect/(b) in between them.	-	-	0.31	0.37	-	**0.75**
		E-B-4	(a) think connecting/(b) in between them will help my company.	-	-	-	-	-	**0.84**
		E-B-5	(a) think connecting/(b) in between them will improve my company’s performance.	-	-	-	-	-	**0.82**
		E-B-6	(a) think connecting/(b) in between them will strengthen my company’s capabilities.	-	-	0.30	-	-	**0.83**
Eigen-value				5.66	5.01	5.00	4.93	4.71	4.58
% of Variance				15.73	13.92	13.90	13.71	13.08	12.72
Cumulative %				15.73	29.65	43,55	57.26	70.34	83.06
Cronbach’s Alpha				0.942	0.912	0.961	0.964	0.934	0.978

NOTE: Extraction Method: Principal Component Analysis. Rotation Method: Varimax with Kaiser Loadings that are 0.30 or less are not shown. ^a^ Rotation converged in 7 iterations.
